# MicroTar: predicting microRNA targets from RNA duplexes

**DOI:** 10.1186/1471-2105-7-S5-S20

**Published:** 2006-12-18

**Authors:** Rahul Thadani, Martti T Tammi

**Affiliations:** 1 Department of Biological Sciences, National University of Singapore, 14 Science Drive 4, Singapore 117543; 2 Department of Biochemistry, National University of Singapore, 8 Medical Drive, Singapore 117597; 3 Karolinska Institutet, Department of Microbiology, Tumor and Cell Biology, Stockholm, Sweden

## Abstract

**Background:**

The accurate prediction of a comprehensive set of messenger RNAs (targets) regulated by animal microRNAs (miRNAs) remains an open problem. In particular, the prediction of targets that do not possess evolutionarily conserved complementarity to their miRNA regulators is not adequately addressed by current tools.

**Results:**

We have developed MicroTar, an animal miRNA target prediction tool based on miRNA-target complementarity and thermodynamic data. The algorithm uses predicted free energies of unbound mRNA and putative mRNA-miRNA heterodimers, implicitly addressing the accessibility of the mRNA 3' untranslated region. MicroTar does not rely on evolutionary conservation to discern functional targets, and is able to predict both conserved and non-conserved targets. MicroTar source code and predictions are accessible at , where both serial and parallel versions of the program can be downloaded under an open-source licence.

**Conclusion:**

MicroTar achieves better sensitivity than previously reported predictions when tested on three distinct datasets of experimentally-verified miRNA-target interactions in *C. elegans, Drosophila*, and mouse.

## Background

MicroRNAs (miRNAs) are a class of endogenous, small regulatory RNA averaging 22 nucleotides in length that mediate the post-transcriptional regulation of messenger RNAs. They bind to target messages in a sequence-specific manner, and induce translational repression or endonucleolytic cleavage. The first two miRNAs, *lin-4 *and *let-7 *were discovered some seven years apart in the worm *C. elegans*, in genetic screens for mutants with disrupted developmental timing [1,2]. There has since been an explosion of interest in the field, and the identification of hundreds of miRNAs in metazoans as disparate as vertebrates, arthropods, nematodes, and viruses [3] has established miRNAs as pervasive regulators of gene expression. For recent reviews, see [4-6].

Functions have only been experimentally assigned to a small fraction of the few thousand known miRNAs [7]. Of the experimental strategies available to investigate miRNA function, stringent genetic tests that link miRNA loss-of-function mutants to misregulated targets, and point mutations in miRNA binding sites to specific phenotypes are impractical on a genomic scale in any animal species [8]. Tissue-culture assays using reporter gene constructs fused to target sequences are an easier alternative, but their reliance on ectopic miRNA expression harbours the danger of measuring what may be a nonphysiological interaction between two molecules with complementary surfaces [9]. Computational approaches are thus likely to remain an important means of studying miRNA targets for the forseeable future, not least as a means of directing wet-lab experiments. These predictions are no doubt hampered by the fact that animal miRNAs – in contrast to plant miRNAs – tend to be only partially complementary to their target mRNAs. This fact, compounded by the small size of these molecules, precludes the use of standard sequence comparison methods.

Several algorithms have been developed to predict miRNA targets in animal species; these are listed in Table 1. A common strategy in several of these programs is to rank target 3' untranslated region (UTR) complementarity by some combination of duplex free energy and/or pairing requirements at the 5' end (seed region) of the miRNA [8]. For instance, TargetScan [10] combines requirements for conserved perfect Watson-Crick pairing at positions 2–8 of the miRNA with estimates of the free energy of isolated miRNA-target site interactions, ignoring initiation free energy. While *in vitro *tests have shown sites containing G:U base-pairs to be functional but impaired [11], recent *in vivo *experiments have demonstrated them to be efficiently downregulated [9]. Taken together with the presence of a G:U base-pair in the seed region of a functional *let-7 *binding site in the *lin-41 3*'-UTR [12], these results make a case for the inclusion of seeds with G:U wobbles in target prediction algorithms.

The PicTar [13,14] algorithm defines seeds as heptamers with Watson-Crick or G:U pairings at positions 1–7 or 2–8 from the miRNA 5' end. It combines seed searches with RNA duplex free energy filters, evolutionary conservation requirements, and a probabilistic scoring mechanism to predict targets that are under combinatorial control by co-expressed miRNAs. However, it makes use of RNAHybrid [15], an algorithm that approximates RNA duplex free energies by discarding intramolecular hybridizations in order to achieve linear time complexity.

Robins *et al*. [16] incorporate mRNA secondary structure computed from 3'-UTRs in their target prediction algorithm, but require perfect Watson-Crick complementarity in the seed site. Furthermore, the use of isolated 3'-UTRs is likely to produce structures very different from the structure of 3'-UTRs in folds that use complete mRNA sequences.

While most of the tools listed in Table 1 are accessible as web services, only miRanda [17] and RNAHybrid are available as downloadable software that can be modified, extended and run on custom datasets. Most listed algorithms also rely on target conservation across two or more species as a filter. While this is necessary to distinguish functional targets from a vast array of candidates, it results in the unavoidable omission of real targets that are not thus conserved.

Here we present MicroTar, an miRNA target prediction program that does not rely on evolutionary conservation. Through the use of the partial complementarity of miRNAs to their target messages, and the predicted free energy of complete mRNA molecules, we are able to address the problem of the prediction of targets that are not conserved across different genomes. Moreover, harnessing the power of parallel computing obviates the need for introducing approximations that discard intramolecular base pairs in estimates of miRNA-mRNA duplex free energy; we thus implicitly incorporate the accessibility of 3'-UTRs in the algorithm. MicroTar source code – available under an open-source licence – and predictions can be accessed at the MicroTar website [18].

## Implementation

### Overview

The MicroTar algorithm is based on the following assumptions:

• miRNA target specificity is determined by a heptameric seed sequence (beginning at the first or second position from the 5' end of the miRNA) that is complementary to sites in mRNA 3'-UTRs

• targets are functional if miRNA-mRNA duplex formation is energetically favourable

**Figure 1:**
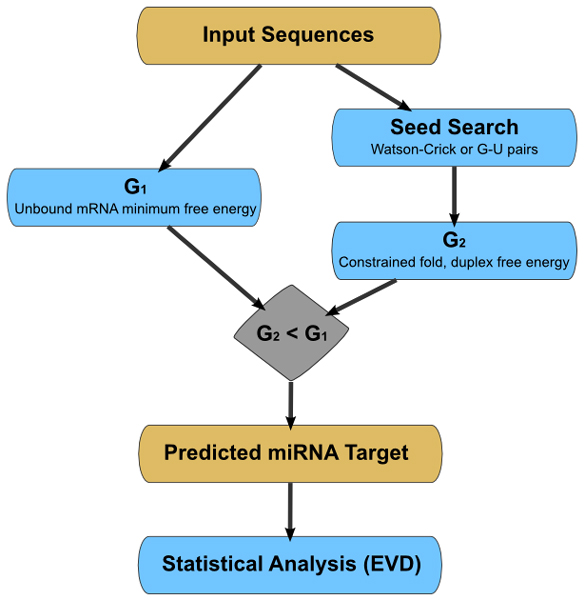
**MicroTar algorithm**. Beginning with a set of fasta-formatted query (miRNA) sequences and target (mRNA) sequences, the MicroTar algorithm predicts the minimum free energy of the each mRNA molecule, searches for seed sites, and performs a constrained fold where each seed match is, in turn, bound in the miRNA-mRNA heterodimer; the output is a list of putative duplexes more stable than free mRNA, along with images of bound and unbound mRNA secondary structure. This result is subsequently subjected to a statistical analysis to determine the significance of each miRNA-mRNA match.

Beginning with a set of fasta-formatted query (miRNA) sequences and target (mRNA) sequences, the MicroTar algorithm predicts the minimum free energy of the each mRNA molecule, searches for seed sites, and performs a constrained fold where each seed match is, in turn, bound in the miRNA-mRNA heterodimer; the output is a list of putative duplexes more stable than free mRNA, along with images of bound and unbound mRNA secondary structure. This result is subsequently subjected to a statistical analysis to determine the significance of each miRNA-mRNA match. Figure 1 presents a schematic overview of this algorithm.

#### Secondary structure prediction

The secondary structure and minimum free energy of the complete unbound mRNA molecule are predicted using the fold routine from the RNAlib library of the ViennaRNA package [19]. This is an implementation of the Zuker & Stiegler dynamic programming algorithm [20]. We denote the predicted free energy of unbound mRNA as *G*_1_.

#### Seed search

Loss-of-function mutation studies have demonstrated the core of miRNA sequence specificity to be a heptameric seed sequence [11], which we define as nucleotides 1–7 or 2–8 at the 5' end of the miRNA. MicroTar searches each mRNA 3'-UTR (or complete mRNA in the absence of annotations) for sites with Watson-Crick or G–U wobble complementarity to this seed sequence; we refer to these hits as seed matches.

#### Constrained fold

For each seed match above, the mRNA is again folded under the constraint that the miRNA seed is bound to its corresponding match. This uses the cofold [21] routine from the RNAlib library. We denote the free energy of the duplex as *G*_2_.

#### Output

**Figure 2:**
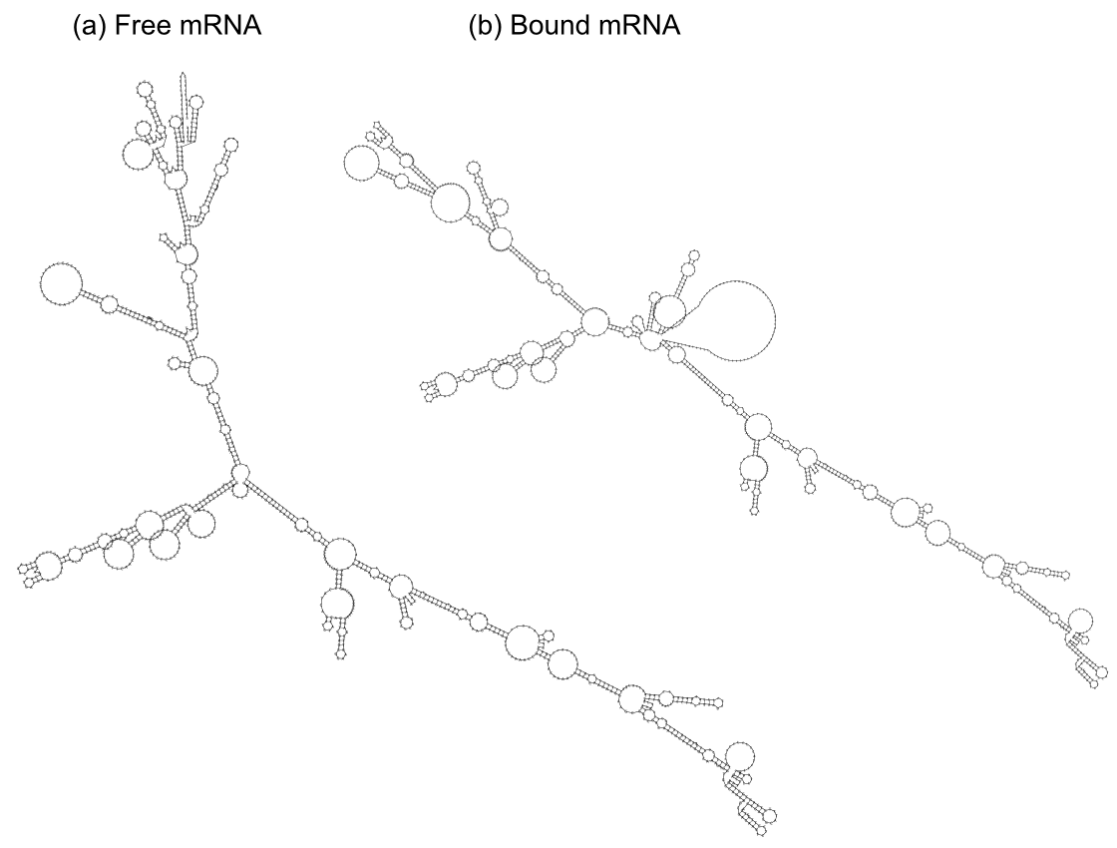
**mRNA secondary structure**. Sample output of the *C. elegans*. cog-1 [GenBank:NM_001027093] mRNA secondary structure before and after binding with the lsy-6 miRNA. Note the changes in global structure, which cannot be approximated using only 3'-UTRs.

The output is a list of all seed matches, along with predicted energies of the unbound mRNA (*G*_1_), putative mRNA-miRNA heterodimers (*G*_2_), the estimated energy of duplex formation (*g *= *G*_2 _- *G*_1_), and optionally, images of the secondary structure of each mRNA before and after miRNA binding (see e.g., Figure 2).

#### Functional targets

Seed matches are considered functional targets if the relevant miRNA-mRNA heterodimer is more energetically stable than free mRNA, i.e., *g *< 0. We then estimate the significance of the prediction using extreme value statistics, much in the fashion of Rehmsmeier *et al*. [15]. This procedure is outlined below.

### Statistical analysis of predicted targets

#### Negative normalized free energy

The occurrence of favourable hybridizations of short miRNAs with long mRNAs can frequently be attributed to chance: the longer the mRNA, the more likely the incidence. In order to eliminate the effect of sequence length on our measure of free energy [15,22], we define the negative normalized free energy



where *m *is the length of the target sequence searched, and *n *is the length of the miRNA.

#### Extreme value statistics

Extreme value distributions (EVDs) are limiting distributions that describe the minimum or maximum of independent random variables [23]. If we consider the miRNA-mRNA duplex energy estimation to be essentially an optimization procedure that produces a minimum, the negative normalized free energy described above is a corresponding maximum, and can be described by an EVD having a distribution function of the form



A transformation then converts this distribution function into a straight line:



By scanning for targets of random miRNA sequences in the mRNA sequences in the dataset, we obtain a set of negative normalized free energies, which we expect will follow an EVD. We then transform the distribution function of the empirical EVD into a straight line, as in Equation 3, and estimate the parameters of the EVD by a linear least squares fit to the line *y *= *mx *+ *c*, obtaining



and

*a *= *cb*.     (5)

We can now compute, for each predicted miRNA-mRNA duplex, a *p*-value, the probability that the same or a more favourable free energy is observed due to chance:



where *a *and *b *are estimated EVD parameters, and *g*_*n *_is the negative normalized free energy from Equation 1 [15].

### Technical details

MicroTar has been written using the C programming language, and makes use of the RNAlib library from the Vienna RNA package [19]. Great care has been taken to make the system suitable for datasets of varying sizes. Sequences are loaded into memory only as required, allowing the handling of virtually any number of sequences. The parallel version uses functions from v2.0 of the Message Passing Interface (MPI).

MicroTar should compile and run under Linux and most flavours of UNIX. It has been tested under Fedora Core 4 & 5 and CentOS 4.4 Linux distributions, on both 32 and 64 bit platforms.

## Results and Discussion

### Validation

**Figure 3:**
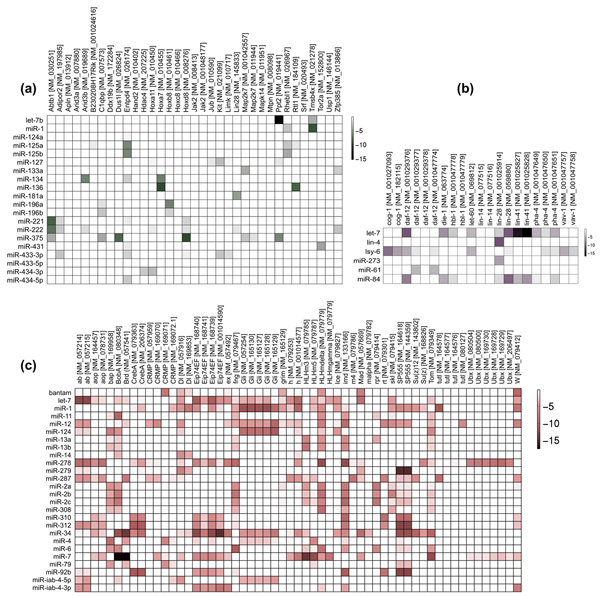
**Energies of predicted miRNA targets**. A density plot of free energies of the most stable predicted miRNA-target duplex for each gene-miRNA pair in (a) mouse, (b) *C. elegans*, and (c) *Drosophila*, with genes along the x-axis and miRNAs along the *y*-axis. A more negative free energy indicates a more stable duplex, relative to its unbound mRNA. Darker colours indicate lower free energies, as shown by the scale in the top-right corner of each sub-figure. White squares indicate no predicted interaction.

We performed a test of MicroTar on three sets of experimentally verified miRNA targets in *C. elegans, Drosophila*, and mouse, from v3.0 of TarBase [7]. miRNA sequences were retrieved from miRBase v9.0 [3]; mRNA sequences from RefSeq entries associated with the corresponding gene entry in the Entrez Gene database. In the absence of 3'-UTR annotations, the entire mRNA sequence was scanned for seed matches by MicroTar. These results are summarized in Figure 3, which shows a density plot of free energies of the most stable predicted miRNA-target duplex for each gene-miRNA pair in the three species.

Furthermore, we compared our predictions to the widely-used PicTar algorithm, which was recently updated and applied to miRNAs in *C. elegans*. This comparison is shown in Table 2, where we note that MicroTar achieves better sensitivity in all three cases. We emphasize that unverified predicted interactions should be viewed as a guide for further experiments and not as false positives. Detailed lists of targets predicted are available as supplementary data (see Additional File 1 – MicroTar target predictions compared to PicTar), and on the MicroTar website [18].

### Duplex energy estimation

At the core of the MicroTar algorithm lies a novel approach to the estimation of miRNA-mRNA duplex energy. Interactions are viewed in a global context by predicting folds for the entire mRNA, rather than just its 3'-UTR or seed match. By allowing intramolecular hybridizations, we implicitly incorporate the accessibility of the 3'-UTR; seed matches in highly inaccessible UTRs are expected to disrupt UTR secondary structure in putative duplexes. Large disruptions in base pairing cannot be compensated for by bond formation during miRNA-mRNA hybridization. This results in a putative duplex with free energy *G*_2 _far greater than that of the unbound mRNA, *G*_1_, and the match is rejected.

### Significance of predictions

**Figure 4:**
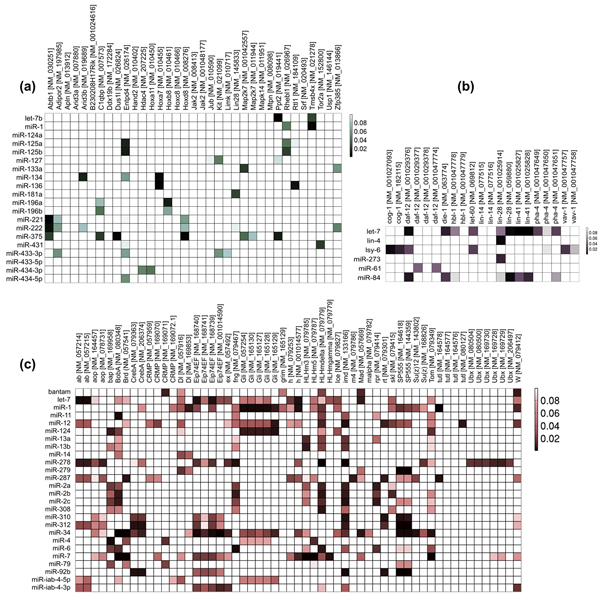
**p-values of predicted miRNA targets**. A density plot of p-values lower than 0.1, of the most stable predicted miRNA-target duplex for each gene-miRNA pair in (a) mouse, (b) *C. elegans*, and (c) *Drosophila*, with genes along the *x*-axis and miRNAs along the *y*-axis. A lower *p*-value indicates a lower probability of the energy of the duplex (or more favourable energies) occurring due to chance alone. Darker colours indicate lower *p*-values, as shown by the scale in the top-right corner of each sub-figure. White squares indicate no predicted interaction, or a *p*-value greater than the cuto3 value of 0.1.

In order to estimate the significance of our predictions, we calculated the p-value for the lowest energy duplex for each miRNA-transcript pair, as derived in Equation 6. The parameters were estimated separately for each species from a distribution computed with random miRNAs. We shuffled miRNAs using the shuffleseq utility from the EMBOSS package [24], ensuring that there were a sufficient number of random sequences for approximately 4000 seed matches in each species. Figure 4 shows these *p*-values in a density plot for each miRNA-target pair, as in Figure 3.

## Conclusion

MicroTar does not rely on evolutionary conservation to filter predicted targets and is able to address the problem of the prediction of targets that are not conserved across different genomes. Parallel computing makes feasible the use of complex energy prediction algorithms on a large scale, and by using estimates of miRNA-mRNA duplex free energy that allow intramolecular pairings, MicroTar implicitly incorporates the accessibility of 3'-UTRs. In tests on three datasets of experimentally verified miRNA targets in *C. elegans, Drosophila *and mouse, MicroTar displays greater sensitivity than previously developed target prediction programs.

## Availability and Requirements

**Project name**: MicroTar

**Project home page**: 

**Operating systems**: Linux, UNIX

**Programming language**: C

**Other requirements**: GNU autoconf/automake

**Licence**: New BSD licence

**Any restrictions to use by non-academics**: None (check ViennaRNA licence, however)

## Authors' contributions

MTT and RT planned the project. RT acquired the data and implemented the algorithm. Both authors prepared and approved the final manuscript.

## Supplementary Material

Additional File 1 **MicroTar target predictions compared to PicTar**. A list of all experimentally verified targets in the three datasets used (*C. elegans, Drosophila *and mouse), with a comparison of those predicted by MicroTar and those found on the PicTar website. Click here for file

## Figures and Tables

**Table 1:** miRNA target prediction tools. A list of current miRNA target prediction tools, with access details. Note that only RNAHybrid and miRanda provide source code for download.

**Program**	**Interface**	**Reference(s)**
**miRanda**	Web access to predictions, downloadable software	[17]
**PicTar**	Web access to predictions	[13,14]
**TargetScan**	Web access to predictions	[10]
**RNAHybrid**	Web submission, Web API, downloadable software	[15]
**MicroInspector**	Web submission	[25]
**DIANA-microT**	Web submission	[26]
**Targetboost**	Web access to predictions	[27]
[Stark *et al*.]	Article supplementary data	[28]
[Robins *et al*.]	Article supplementary data	[16]

**Table 2:** MicroTar target predictions compared to PicTar. A comparison of MicroTar and PicTar prediction results on three datasets of experimentally verified miRNA targets; MicroTar achieves better sensitivity in all three cases.

**Program**	**Species**	**Targets Predicted **(TP)	**Targets in Dataset **(TP + FN)	**Sensitivity **TP/(TP + FN)
MicroTar	*D. melanogaster*	39	63	0.62
	*C. elegans*	8	13	0.62
	*M. musculus*	24	43	0.56
PicTar	*D. melanogaster*	35	63	0.56
	*C. elegans*	7	13	0.54
	*M. musculus*	15	43	0.35
